# Microdosimetry performance of the first multi-arrays of 3D-cylindrical microdetectors

**DOI:** 10.1038/s41598-022-14940-1

**Published:** 2022-07-18

**Authors:** Diana Bachiller-Perea, Mingming Zhang, Celeste Fleta, David Quirion, Daniela Bassignana, Faustino Gómez, Consuelo Guardiola

**Affiliations:** 1 Université Paris-Saclay, CNRS/IN2P3, IJCLab, 91405 Orsay, France; 2 Université Paris-Cité, IJCLab, 91405 Orsay, France; 3 Instituto de Microelectrónica de Barcelona (IMB-CNM, CSIC), 08193 Barcelona, Spain; 4 Departamento de Física de Partículas, Universidad de Santiago de Compostela, 15782 Santiago de Compostela, Spain

**Keywords:** Applied physics, Techniques and instrumentation, Radiotherapy

## Abstract

The present work reports on the microdosimetry measurements performed with the two first multi-arrays of microdosimeters with the highest radiation sensitive surface covered so far. The sensors are based on new silicon-based radiation detectors with a novel 3D cylindrical architecture. Each system consists of arrays of independent microdetectors covering 2 mm$\times$2 mm and 0.4 mm$\times$12 cm radiation sensitive areas, the sensor distributions are arranged in layouts of 11$\times$11 microdetectors and 3$\times$3 multi-arrays, respectively. We have performed proton irradiations at several energies to compare the microdosimetry performance of the two systems, which have different spatial resolution and detection surface. The unitcell of both arrays is a 3D cylindrical diode with a 25 $\mu$m diameter and a 20 $\mu$m depth that results in a welldefined and isolated radiation sensitive micro-volume etched inside a silicon wafer. Measurements were carried out at the Accélérateur Linéaire et Tandem à Orsay (ALTO) facility by irradiating the two detection systems with monoenergetic proton beams from 6 to 20 MeV at clinical-equivalent fluence rates. The microdosimetry quantities were obtained with a spatial resolution of 200 $\mu$m and 600 $\mu$m for the 11$\times$11 system and for the 3$\times$3 multi-array system, respectively. Experimental results were compared with Monte Carlo simulations and an overall good agreement was found. The good performance of both microdetector arrays demonstrates that this architecture and both configurations can be used clinically as microdosimeters for measuring the lineal energy distributions and, thus, for RBE optimization of hadron therapy treatments. Likewise, the results have shown that the devices can be also employed as a multipurpose device for beam monitoring in particle accelerators.

## Introduction

Beam characterization is essential in particle accelerator monitoring, commissioning and quality assurance (QA) due to the high precision required on the beam delivery for different applications^1^. In particular, in radiotherapy, tumors must be irradiated with millimeter precision, which requires a high accuracy of the beam monitoring for verification of the treatment planning. Errors in the beam delivery, planning, or in the patient set-ups can severely impact the healthy tissues surrounding the target volume. Therefore, different types of sophisticated detectors or imaging advanced techniques are used to facilitate beam quality assurance and accurate dose delivery^2^. QA involves also the beam control in novel delivery systems, e.g., for intensity-modulated radiation therapy (IMRT), which have a higher complexity and limited technologies.

In this context, treatments based on hadron therapy (HT) are rapidly expanding worldwide^3,4^. These are advanced radiotherapy modalities that make use of proton and carbon ions to preserve healthy tissues as much as possible, which is fundamental in radiation medicine. HT provides a highly conformed and uniform dose to the tumor while sparing healthy tissues. It is mainly possible due to the fact that proton and carbon ion beams release low amounts of energy at the tissue entrance, and a maximal energy at the Bragg Peak position, where the particles dramatically lose their energy. Hence, HT achieves very high dose conformity around the tumor, allowing a better protection of the surrounding tissues, which is particularly critical for tumors localized near organs at risk and pediatric cancers^5^. Despite HT advantages, some normal tissue toxicities are beginning to be reported^6–9^, which could be avoided by developing new technologies as microdosimeters. These toxicities may lead to severe side effects as influencing cognitive function in children^8^, functionality of organs, or even secondary cancers^9^. It is particularly important in pediatric and young patients, since they are prone to carcinogenic effects and their life expectancy is longer than the latent period of the secondary effects^10,11^. These issues can be partially due to the fact that ions deliver more energy per unit of track length (higher linear energy transfer, LET) than conventional radiotherapy sources^12^. If high-LET spots are not properly controlled, they may generate collateral damages, e.g., acute and late effects, and even secondary cancer induction. Therefore, the LET is the major physical descriptor of the biological damage at macroscopic level, but at subcellular level its stochastic nature prevails and the lineal energy distributions turn into the appropriate parameter for microdosimetry characterization^13–15^. The lineal energy (*y*) is defined as the ratio between the deposited energy by a single event into a given microscopic volume and the corresponding mean chord length (or mean path length, as usually referred to in the field of particle therapy, ${\bar{l}}$) of that irradiated volume^16^. Quantifying LET with microscopic resolution would allow us to optimize the treatments either removing high-LET spots from critical structures or focusing high-LET regions into the target^17–19^. Although LET can be simulated rather than measured, for the particular case discussed herein, i.e., low energy protons, the dose averaged lineal energy, $y_{D}$, is a good approximation of the simulated $LET_{D}$^20,21^.

The measurement of microdosimetric distributions require the determination of the energy imparted by the incident particles in microscopic volumes. In the beginning, the gold standard in microdosimetry was based on Tissue Equivalent Proportional Counters (TEPCs)^14^. Currently, silicon-based radiation detectors have come up as feasible microdosimeters due to the well-established microfabrication processes that may provide well-defined customized radiation sensitive micro-volumes^21–27^. In this context, the Rosenfeld’s group and co-workers have contributed significantly to microdosimetry verification in the last two decades^28,29^. The first three generations of their SOI microdosimeters were based on planar p-n junctions. Their fourth generation (“bridge”) was based on planar p-n junctions placed on top of 3D rectangular parallelepiped (RPP) shape sensitive volumes fabricated by Deep Reactive Ion Etching (DRIE) with fully etched out surrounding silicon. In the last fifth generation^28^, a 3D detector technology was suggested and used for fabrication of cylindrical SVs (“mushrooms”) of SOI microdosimeter in Norway and tested with different ions^24,30^, similar to those fabricated with 3D detector technology and characterized by authors in^31^. In that structure, the sensitive volumes were separated into arrays, whose signal was read jointly, thus, reducing the spatial resolution. Recently, they have compared their last generation of microdetectors with a new mini-TEPC^32^. Another interesting microdosimeter was proposed by Agosteo *et al.*^33,34^, based on a monolithic silicon device consisting of a matrix of cylindrical diodes (2 $\mu$m thick, 9 $\mu$m diameter) coupled to a residual energy measurement stage (500 $\mu$m thick). It has been recently compared with reference TEPCs^35,36^. However, none of the existing systems (commercial or experimental) have spatial resolution along the transverse beam direction and of a data acquisition adaptation for clinical conditions. In order to improve the current silicon-based microdosimeters, a new 3D-cylindrical architecture of silicon-based radiation detectors was designed in 2012 and eventually manufactured in 2015^31,37^ by using micro manufacturing techniques optimized for 3D detectors that had been developed by Pellegrini *et al.* during the previous decade in the Radiation Detectors group of the National Center of Microelectronics (IMB-CNM, CSIC), Spain^38–40^. Compared to planar silicon detectors, the 3D architecture reduces the loss of charge carriers due to trapping effects, the charge collection time, and the voltage for full depletion^41–43^. Additionally, the well delimited and truly isolated radiation sensitive volume (SV) of the 3D cylindrical architecture are able to mimic the microscopic sizes of mammalian cells. The sensors have already shown a good performance in microdosimetry at both carbon therapy^44^ and low energy protons facilities^45^. Details about the fabrication processes, electrical simulations and charge collection efficiency studies of the devices are described somewhere else^42,43,46,47^. Based on these sensors, we have recently created an improved generation of microdetector arrays, with individual 3D-cylindrical detectors (pixel type configuration), covering from millimeters to centimeters of radiation sensitive area. The SV underlying these arrays has the following features: 20 $\mu$m thickness, 25 $\mu$m diameter, and 200 $\mu$m pitch (distance from center to center between two adjacent microdetectors). In this work, we use two novel array layouts: (i) a two dimensional array of 11$\times$11 independent microdetectors covering a 2 mm$\times$2 mm radiation sensitive area, and (ii) A linear array in which 3$\times$3 microdetectors are grouped together with a total surface of 0.4 mm$\times$12 cm. To the best of our knowledge, this is the largest radiation sensitive surface covered with microdosimeters so far. Likewise, we have improved the charge collection efficiency (CCE) of the devices in this last generation, as it was reported in^47^. This plays a relevant role for the data collection and interpretation, since an inhomogeneous CCE produces that events from different regions in the volume generate different pulse heights, which biases the reconstruction of the energy imparted per event. Prieto-Pena *et al.* showed that the CCE can be considerably less than 100 % in various regions of the detector, especially near or inside the collecting electrodes, where the electric field is null and the charge carriers move only by diffusion^48^. In particular, the improved CCE of the SV used in this work ranges between 100 % and 90 % for radial distances up to 10.75 $\mu$m (from the center of the sensors) and then it rapidly decays between 10.75 $\mu$m and the detector edge^47^. This CCE correction factor has been taken into account and included in the corresponding simulations to precisely compare them to the experimental results.

Additionally, the microdetector systems were assembled to a specific multichannel data-acquisition (DAQ) system for spectroscopy. Two dedicated daughterboards, as well as special pitch-adapters to connect the microdetector arrays with the electronics, were designed and manufactured to reduce the overall noise, allowing to measure proton LETs as low as those occurring at shallow depth in proton therapy, namely, $\sim$ 2 keV/$\mu$m.

The present paper reports on the first comparison of experimental microdosimetry of 3D-cylindrical microdetector arrays from 2 mm$\times$2 mm to 0.4 mm$\times$12 cm at clinical-equivalent fluence rates in proton beams with the highest spatial resolution laterally so far. Results showed that this new architecture can be used not only for microdosimetry characterization, but also for beam monitoring.

## Methods

Two microdosimeter arrays with different layouts but using the same elementary detectors, have been designed, fabricated, and characterized by performing irradiations with proton beams. This section describes: (i) the two microdosimeter systems developed, (ii) the experimental irradiation setup used, and (iii) the Monte Carlo simulations performed to compare the experimental results with those simulated.

### Microdosimetry systems

**Figure 1:**
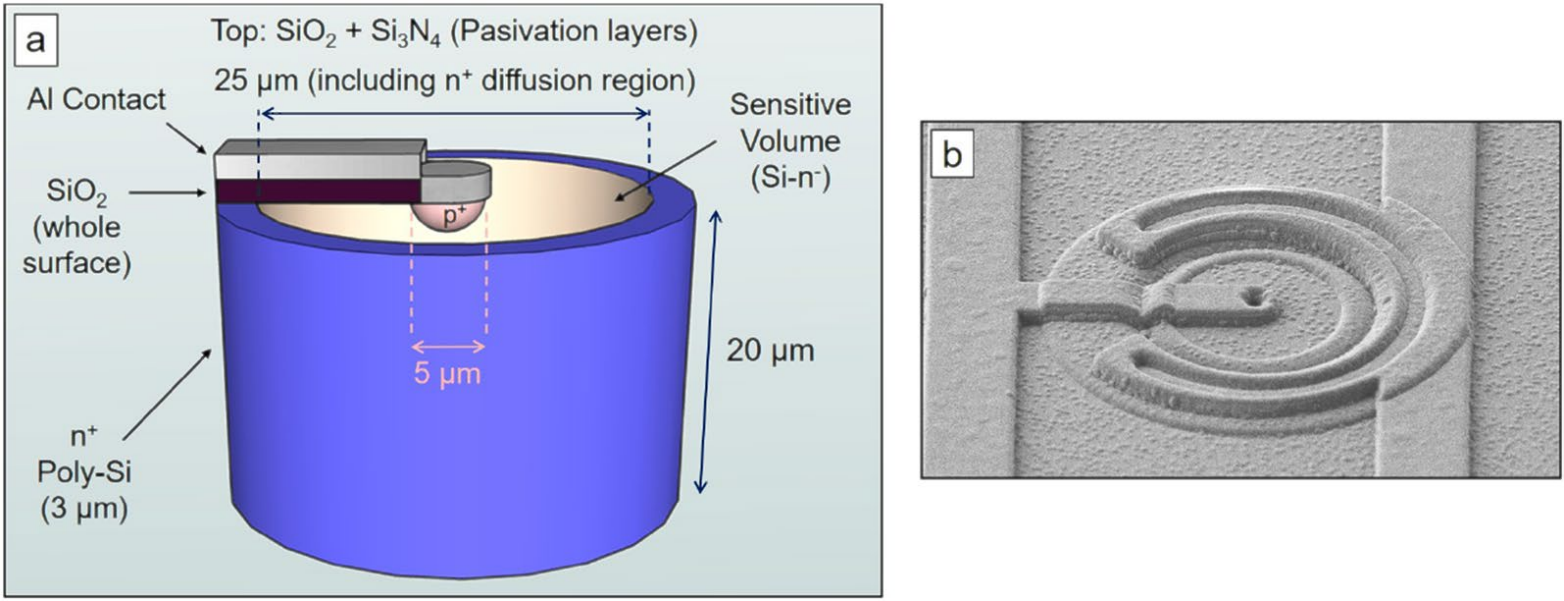
(**a**) Sketch of a 3D cylindrical microdetector (unitcell). (**b**) SEM image of the top surface of a microdetector.

The microdosimetry systems are based on arrays of individual 3D cylindrical silicon microdetectors. The structure of each microdetector (unitcell) is shown in Fig. 1a. It has a diameter of 25 $\mu$m and a thickness of 20 $\mu$m. Figure 1b shows a scanning electron microscope (SEM) image of an individual microdetector.

The microdetectors used in this work belong to the second and third improved microfabrication runs with respect to those studied previously^31,42–44,46,48^. They are fabricated on silicon-on-insulator (SOI) wafers from IceMOS Technology^49^. The sensitive volume (SV) of the detector is manufactured in high resistivity (> 3 k$\Omega \cdot$cm) <100> n-type doped Si, with 1 $\mu$m of buried SiO$_{2}$ and 300 $\mu$m of low-resistivity <100> Si as support. The whole manufacturing process has 122 microfabrication steps, but only the main stages are described here. First, the p$^{+}$-electrode is fabricated by ionic implantation of boron. Second, around the 25 $\mu$m-diameter cylinder, a 3 $\mu$m trench is etched, filled with polysilicon (poly-Si), and then doped with phosphorus using POCl$_{3}$. The P diffuses towards the sensitive volume, creating an n$^{+}$ region by diffusion that will act as ohmic contact. Third, a TEOS-based oxide is deposited over the entire surface of the device to create an insulating layer over the phosphorous-doped polysilicon. Fourth, aluminum strips are deposited to connect the n$^{+}$-electrode to a common contact and the central p$^{+}$-electrode to a channel of the readout chip (ROC) to read out the collected charge. Finally, a SiO$_{2}$/Si$_{3}$N$_{4}$ passivation bilayer is deposited over the whole surface. The final SV thickness was (19.86 ± 0.04) $\mu$m. The uncertainty of the SV thickness was calculated by measuring five independent silicon wafers where the set of microdetectors had been manufactured. Thickness values were carefully quantified during the manufacturing process with an optical method (reflectometry with NanoSpec 6100) and at the end of fabrication processes with the focused ion beam technique. More information about the fabrication process has been already reported in the literature^42,43,46,50^.

**Figure 2:**
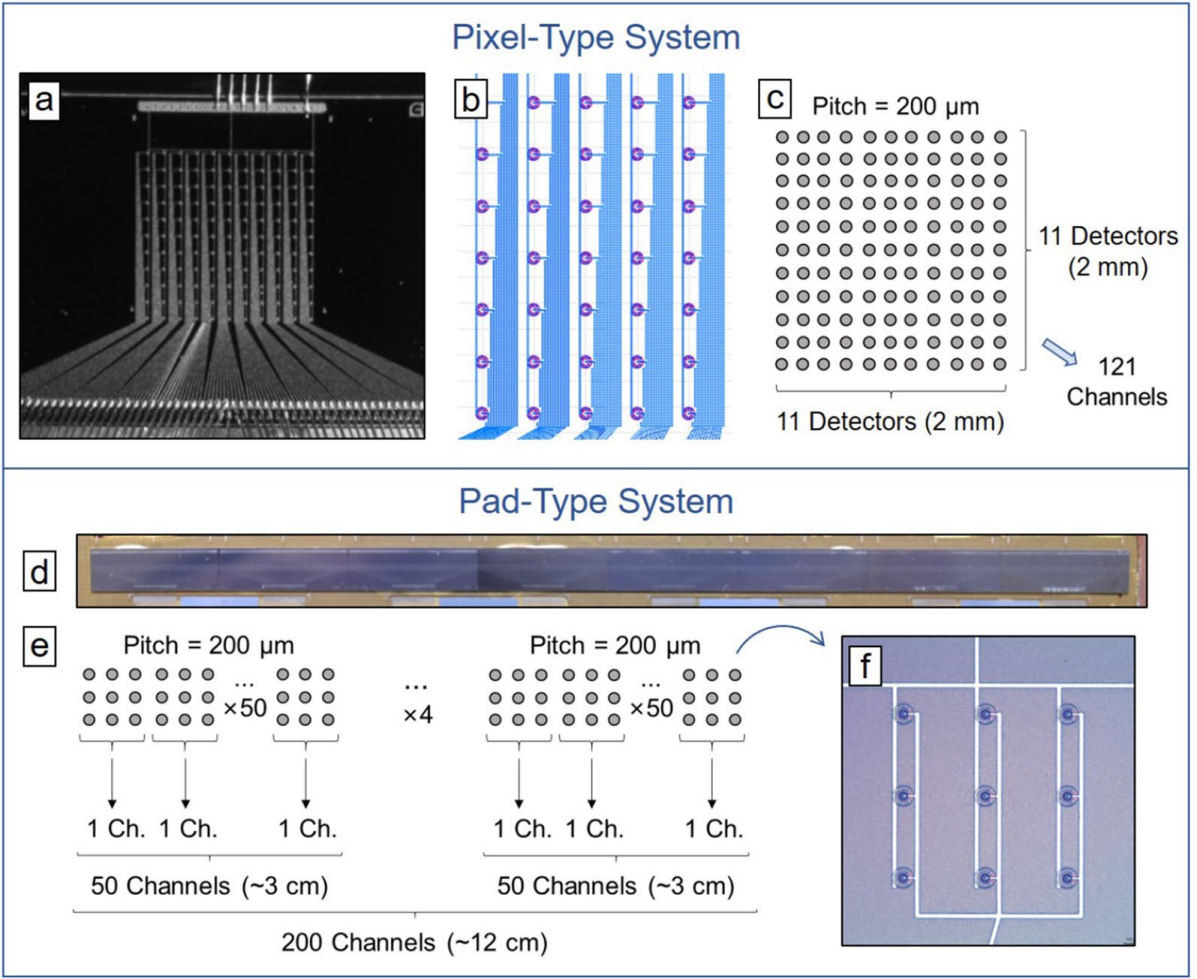
(**a**) Optical image of the 11$\times$11 pixel-type array. (**b**) Detail of the pixel-type layout: each detector is connected to a ROC channel. (**c**) Sketch of the pixel-type array. (**d**) Photograph of the eight pad-type arrays stacked laterally covering a 12 cm length. (**e**) Sketch of the pad-type array. (**f**) Optical image of one of the 3$\times$3 cells.

The two systems studied here are based in two different layouts of the microdetectors (Fig. 2), although the distance between individual detectors (pitch) is 200 $\mu$m in both cases. These two systems offer different possibilities in terms of spatial resolution, detection surface, and accumulated statistics (which will determine the measurement time):

1. Pixel-type system (Fig. 2, top): The array contains 11$\times$11 microdetectors covering 2 mm $\times$ 2 mm. Each microdetector is connected to an individual channel of the ROC in order to analyze the output voltage pulse heights individually. Therefore, there is one ROC with 121 channels connected. The readout strips of the individual SVs (organized in 11 linear arrays with 11 individual SVs in each array) and the corresponding ROC are interconnected by using the wire-bonding technique with aluminum wires.
2. Pad-type system (Fig. 2, bottom): This system is based in arrays containing 3$\times$3 cells. Figure 2f shows on of these 3$\times$3 cells, the 9 microdetectors in each cell are connected to the same readout channel in the ROC. The system has 8 arrays stacked laterally, each array contains 25 3$\times$3 units, covering a total area of 0.4 mm $\times$ 12 cm, the 8 arrays can be observed in Fig. 2d. To obtain a quasi-continuous sensor piece (Fig. 2d), each array was first diced with a diamond micro-saw keeping a distance of (100 ± 10) $\mu$m between its edge and the closest microdetector, in order to keep a pitch of 200 $\mu$m between the last unitcell of one array and the first one of the adjacent array. Thus, the resulting stacked multi-array covers a 12 cm radiation sensitive region in one direction. This system has 4 ROCs, each ROC is used for 2 lateral stacked arrays, therefore, 50 channels (number of 3$\times$3 cells in two arrays, Fig. 2e) are connected per ROC (i.e., 200 channels in total). Since the 3$\times$3 cell size is larger than the ROC pitch, a pitch-adapter (PA) component was required to connect the 3$\times$3 cell to the ROC. Thus, an additional set of new tailored PAs was carefully designed to avoid extra noise and manufactured to connect those multi-arrays with the respective ROCs.

The readout electronics of each system consists of two printed circuit boards (PCBs): a daughterboard that contains the ROCs and the arrays of 3D microdetectors (a different PCB for each system), and a motherboard (the same for both systems) that includes the data-acquisition (DAQ) system. Both PCBs are connected by a 50 cm I/O cable with dual ended connectors, in order to place the motherboard far away of the beam and thereby avoiding potential radiation damage. All the boards are enclosed in independent tailored Faraday cages. The DAQ system consists of a multichannel readout electronics (protected by a utility model) that is able to handle multiple ROCs, where the microdetectors are connected, which allows us to scale the number of microdetector arrays. The detector pads are connected through direct aluminum wire-bonds to the ASIC pads, with an average wire length of less than 5 mm. The DAQ system (which communicates with the computer via Ethernet) controls the acquisition, receives the data, handles the trigger and the data monitoring, and it requires just one small power supply for functioning, making it portable. The system is connected via Ethernet to a PC host where the acquired data are stored. It is controlled by means of a graphical user interface (GUI) in communication with a field-programmable gate array (FPGA) with an embedded processor. The analogue data are digitized, as well as the trigger input signal, with a data transfer rate of 100 Mbps.

The acquired data are stored in HDF5 format and the data analysis can be performed immediately after the irradiation by means of an in-house Python code that displays, among others, the energy spectra and the microdosimetry 2D maps. The overall experimental methodology for the data analysis follows the radiation detection principles detailed by Knoll^51^ and the microdosimetry concepts described by the founders of the microdosimetry theory, Rossi and Zaider^52^. The microdosimetric quantities presented in this work are related to silicon, but they could be easily converted to water (tissue equivalent) by using the density and stopping power of both materials^53^.

### Irradiation setup

**Figure 3:**
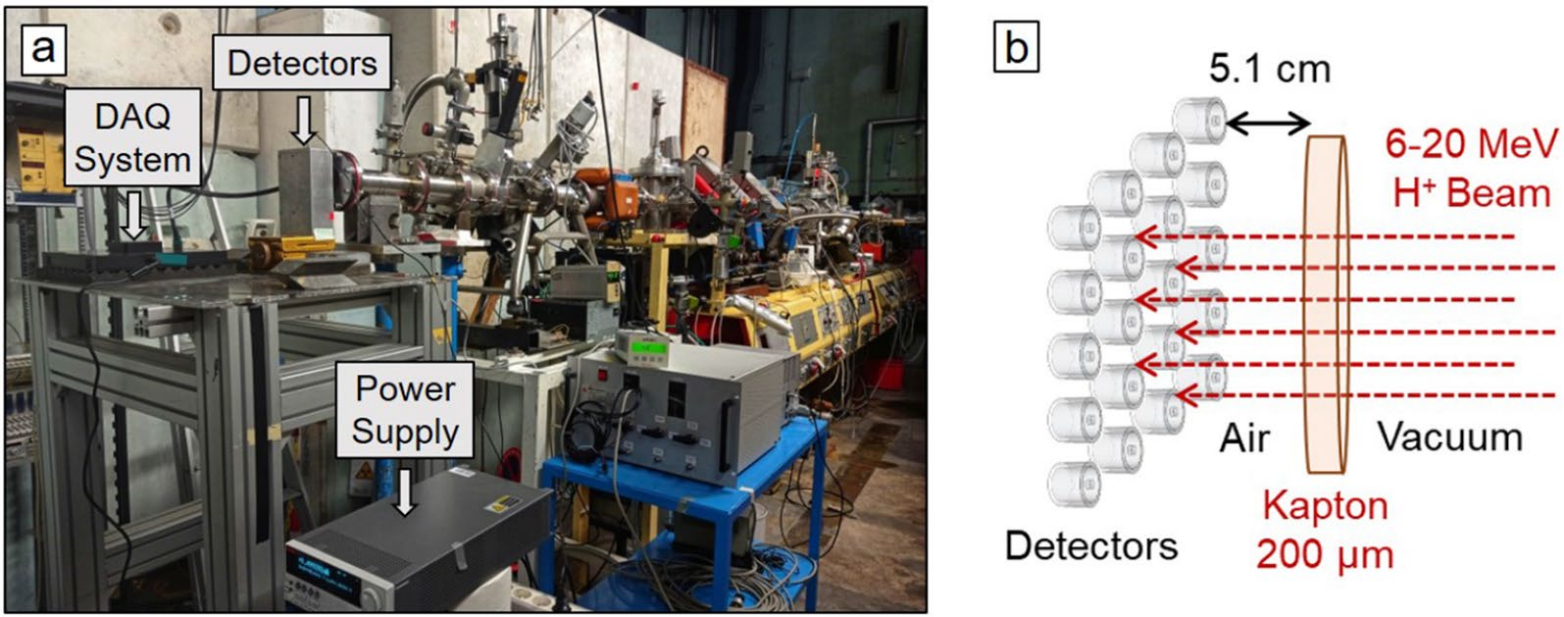
(**a**) Experimental setup used for the irradiations at the beamline 410 of ALTO. The distance between the front face of the detectors and the exit window of the beamline was 5.1 cm. (**b**) Sketch of the irradiation configuration.

Irradiations were performed at the 410 beamline of the Accélérateur Linéaire et Tandem à Orsay (ALTO) facility using a 14.5 MV Van der Graaf tandem accelerator. Proton beams with energies ranging between 6 MeV and 20 MeV were used to irradiate areas covering a few (2-4) cm$^2$ (maximum field size obtained in the exit window). The beam energies between 6 MeV and 20 MeV have been chosen in order to cover practically the full dynamic range of the systems, since the values of the energies deposited into the SV of the detectors vary between 382 keV and 96 keV for this energy range. The beam currents (of the order of 100-200 pA) and the irradiation areas were adjusted to have ion fluence rates of $\sim \,10^8$ cm$^{-2}$ s$^{-1}$, which correspond to the clinical values used for conventional hadron therapy. The irradiation times were of the order of a few minutes to accumulate enough statistics. The microdosimetry systems were placed at the beam exit as shown in Fig. 3a. Between the proton beams and the front face of the detectors there was a 200 $\mu$m-thick kapton and (5.1 ± 0.1) cm of air (Fig. 3b).

### Monte Carlo simulations

Simulations were performed with the GATE (version 9.1) open-source platform^54^, which is a Geant4-based Monte Carlo (MC) code. They were used to compare the pulse-height spectrum measurements. The irradiation configuration as well as the microdetector features described above were simulated. The Physics lists and parameters recommended by the GATE collaboration for proton therapy applications were used, namely the Binary Cascade (BIC) model for the hadronic interactions adding the low-energy electromagnetic processes. These parameters were considered with the GATE builder QGSP$\_$BIC$\_$HP$\_$EMZ^55^. We implemented range cuts of 10 mm, 1 $\mu$m, and 0.5 and 0.2 $\mu$m for all the particles in the “world” (air), SV, and passivation and TEOS layers geometries, respectively.

The proton source was modeled as a so-called general particle source (GPS), which is a G4/GATE predefined option for particle source generation. The energy beam spectra were characterized as Gaussian distributions (from 6 to 20 MeV). The standard deviations for each case were ranged over 0.1 MeV to 1.5 MeV in all the cases to find the best fittings since those deviations have been found previously in this tandem (due to uncertainties associated to different reasons, e.g., tidal deformations from quadrupole contributions, parasitic currents in vacuum chamber, RF frequency and magnet temperatures changes, etc).

The MC simulations were run by using the parallel computing platform developed in GATE and remotely via a Tier1 computation cluster (CC-IN2P3) with a hundred of servers with X86_64 processors in the Linux system. The number of simulated primary protons was $10^{10}$ for each energy. The total energies deposited into the SV of the 3D cylindrical microdetector were recorded. Then, they were treated to account for the CCE dependence on the entry point of the particle trajectory to the SV as follows: the energy spectra were converted into a list of events, a random position in a circle of a 12.5 $\mu$m radius centered in the sensitive volume was assigned for each event. The CCE correction factor for each point was applied by using the measured CCE described by Bachiller-Perea *et al.*^47^ as a function of the distance to the center of the SV. Finally, the energy spectra were reconstructed and compared to the experimental data. Consequently, the lineal energy distributions were calculated by dividing the energies spectrum histograms by the mean path length of the particles in the SV, i.e., 19.86 $\mu$m, which corresponds to the silicon thickness, since we are irradiating perpendicularly to the front face of the microdetectors (Fig. 3b).

## Results

In this section we present, first, the energy calibration of both microdetector systems and the deviations between the individual channels of the ROCs. Secondly, we characterize the proton beam intensity yielded with both systems. Finally, we detail the microdosimetry measurements carried out with them. Four representative beam energies have been chosen to show and discuss the results: 6 MeV, 8 MeV, 10 MeV, and 18 MeV.

### Energy calibration

The calibration allows us to monitor the detector performance. In principle, we assume a linear proportionality between the energy deposited in the silicon microdetectors and the corresponding electronics output voltage pulse height^56^. Due to the small size of the SVs ($<\,10^{-5}$ mm$^{3}$), the extremely low statistics collected with radioactive calibration sources makes unfeasible a proper calibration with them. Therefore, we performed the energy calibration of the systems by means of irradiations with protons at the ALTO facility.

**Figure 4:**
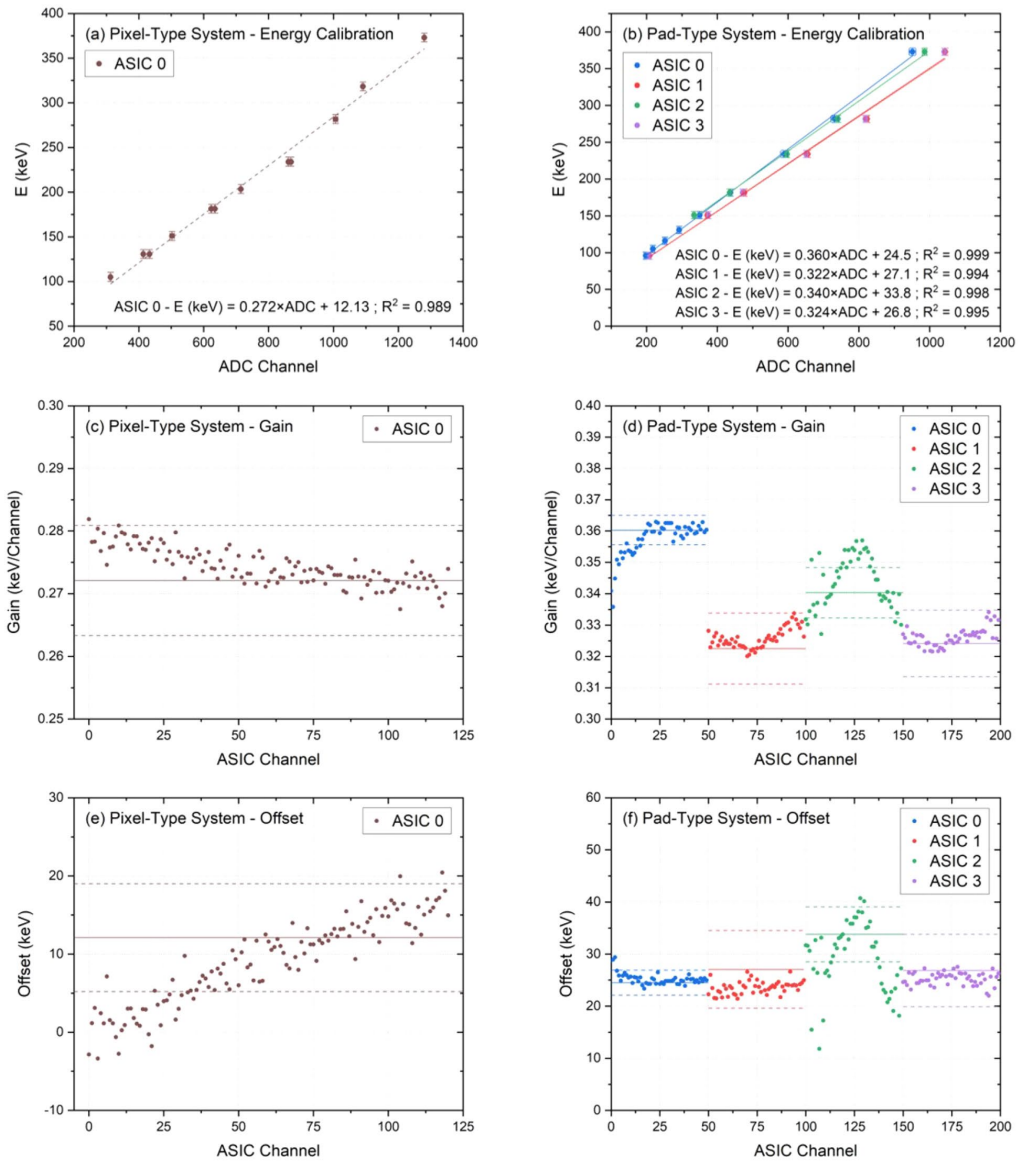
(**a**), (**b**) Energy calibration curves for the pixel- (**a**) and pad-type (**b**) systems. The deposited energies calculated with SRIM simulations were fitted to the ADC channels obtained from the experimental spectra with a linear model. (**c**)–(**f**) Gain and offset values distribution for all the connected channels in each system. The solid lines indicate the mean values obtained with the full ROC energy calibration, and the dashed lines correspond to the uncertainty limits of those values. Channels belonging to different ROCs are displayed in different colors.

On a first stage, we performed a global energy calibration for each readout chip (ROC or ASIC) of the system, considering all the channels connected to the ROC. We fitted the experimental values obtained in analog-to-digital converter units (ADC channels) to the values calculated with Monte Carlo simulations as follows: first, we record the experimental energy spectra obtained when irradiating with the mono-energetic proton beams for both microdetector systems, and we calculated the ADC channel corresponding to the centroid of the main peak in the spectra. Second, the energies deposited by the protons in the SV of the detectors were calculated with the SRIM code (Stopping and Range of Ions in Matter^57^) taking into account the setup and detector geometries described above, a good agreement was found with Geant4 simulations. Then, the most probable imparted energies were matched with the corresponding most probable ADC channels. Hence, for proton beams between 6 MeV and 20 MeV, we obtained values of energies deposited into the SV between 382 keV and 96 keV, respectively. Figure 4a and b show the energy calibrations, including the gain (slope) and offset (intercept) values, obtained for the 11 $\times$ 11 pixel-type system (a) and for the four ROCs of the 3$\times$3 pad-type system (b). The linear regression fit used to correlate both ADC and energy values shows a very good correlation value (R$^2$ $\ge$ 0.98) for all the ROCs in both cases, which confirms the linear pulse-height response in the energy range studied in this work.

On a second stage, we have studied the deviation between the individual ROC channels and the global energy calibration in order to characterize the behavior of the systems. The previous linear fits give us the gain and offset for each ROC, whose mean values and uncertainty limits are represented in Fig. 4c–f with solid and dashed lines, respectively. We have performed the same energy calibration process previously described for each connected channel. Figure 4c–f display (with points) the distribution of the gain and offset values for all the connected detector channels in each system (121 in the pixel-type system, and 50 in each ROC of the pad-type system, as seen in Fig. 2). The maximum deviation between the gain value of each channel and the global ROC calibration was of 3.6 % and 6.7 % for the pixel- and pad-type system, respectively. It is worth noting that the gain may lightly change with the operating conditions.

**Figure 5:**
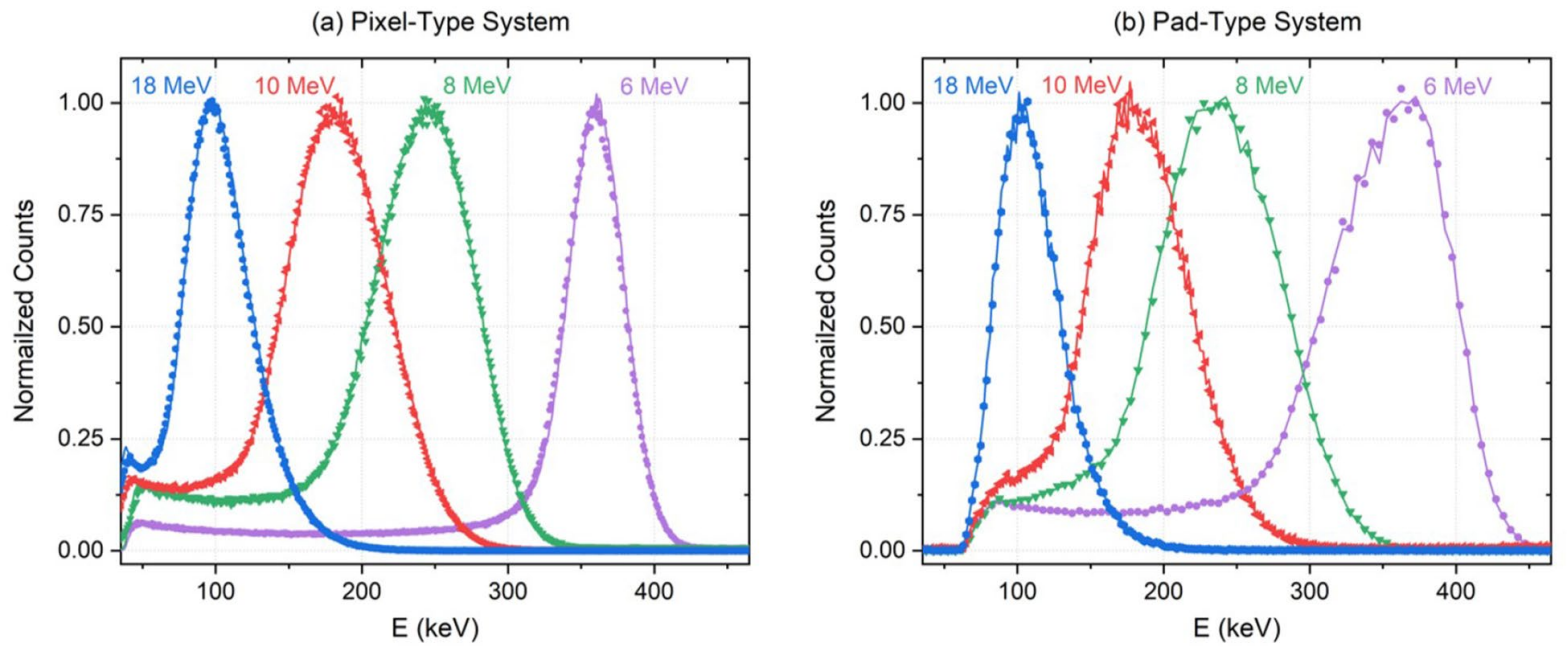
Experimental energy spectra obtained under proton irradiation at four different energies for both systems using the calibration per chip (solid lines) and the calibration per channel (points).

**Figure 6:**
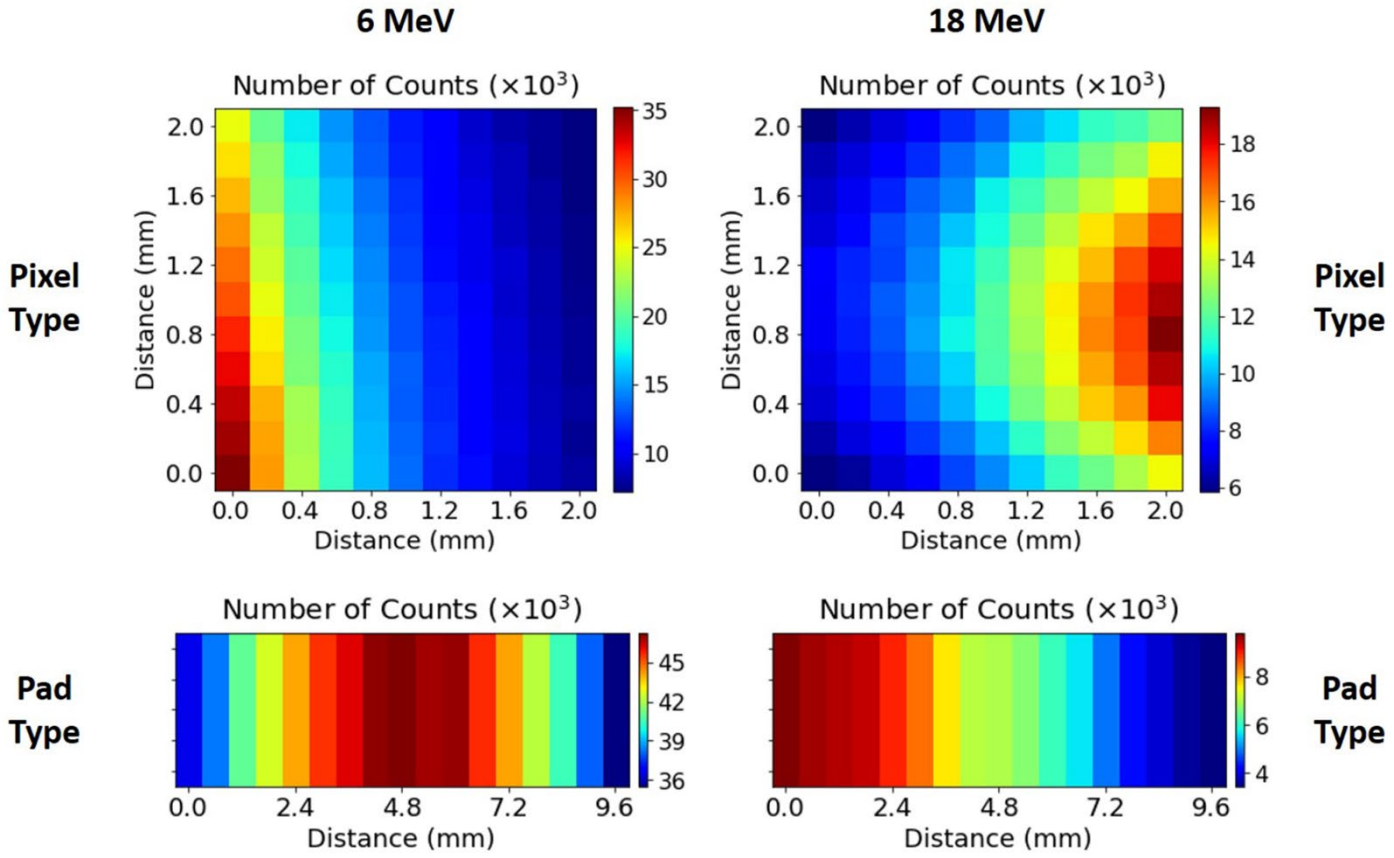
Number of counts measured with 6 MeV (left) and 18 MeV (right) proton beams. Top: pixel-type system. Bottom: 1 cm representative region of the pad-type system (not to scale).

Finally, we have compared the experimental spectra obtained (with proton beams at four different energies) with both systems when converting the ADC channels to energy by using the two calibration modes (Fig. 5). In Fig. 5, the spectra obtained applying the calibration per channel are represented with points, while the spectra applying the full chip calibration are represented with solid lines. The spectra obtained with the pixel-type system are smoother because this system has accumulated a higher total number of counts during the measurements presented here (as can be estimated from Fig. 6 by multiplying the counts per channel by the number of irradiated channels). The results show that the deviation between channels is negligible, since we obtain practically the same energy spectra applying both calibration modes. This proves that a global energy calibration for each ROC in both types of systems can be applied, without necessity of applying a calibration per channel.

These results corroborate that the dynamic range of the system has a linear behavior and is suitable for the deposited energy ranges found at the Bragg peak and distal edge positions, which are fundamental for clinical applications. Hereinafter, the energy calibrations displayed in Fig. 4a, b will be used to analyze the microdosimetry measurements carried out under proton beam irradiation and described in the following sections.

### Beam intensity characterization

Figure 6 shows, as a figure of merit, the total number of counts collected by each of the 11$\times$11 detectors of the pixel-type system (top) and by each 3$\times$3 unit of the pad-type system (bottom), for the lower (6 MeV) and one of the highest (18 MeV) energies used for both systems. In the case of the pixel-type system we have a 2D distribution covering a surface of 2 mm$\times$2 mm with a spatial resolution of 200 $\mu$m in both dimensions (distance between adjacent detectors). With the pad-type system, we can measure along a 1D distance up to 12 cm (in Fig. 6 only a 1 cm representative region is shown) with a resolution of 600 $\mu$m.

These experimental 2D maps of the total number of collected counts prove how these two systems allow us to characterize the spatial dependence of the beam intensity with resolutions of 200 $\mu$m and 600 $\mu$m in two or one dimensions respectively. As it can be observed, the center of the beam changed for different energies (the detector was not moved between measurements). It is also noticeable that the beam distribution presented a diverging and not uniform ellipsoidal shape ($<\,1.5\,$cm beam size), which is not well characterized. This is due to the fact that the 410 beamline at ALTO is used as a multipurpose line, not optimized to work with an homogeneous beam. For example, the magnetic quadrupoles for addressing the beam size are still managed by a manual system. This has a direct impact on the beam tails and halo, which, in turn, is reflected in the heterogeneity of the counting maps.

For the pixel-type configuration (Fig. 6, top), a crosswise gradient is observed in the beam intensity into the 2 mm $\times$ 2 mm area as 6 MeV protons are delivered. Depending on the position, differences in the beam intensity up to a factor of 4 have been found. In contrast, we can clearly identify the ellipsoidal shape of the 18 MeV beam impinging into the same array. This is due to the fact that when the proton beam energies delivered are changed, the optical beam characteristics are also modified, which impact in the final observed counting map. Since the pad-type system covers a distance of several centimeters along one axis, we can observe the beam intensity gradient (with a Gaussian shape) in the 1 cm area irradiated for both energies (Fig. 6, bottom). Another difference between both systems is the acquisition time necessary to accumulate the same statistics (number of counts), since in the pad-type system we have 3$\times$3 detectors connected to each channel, we need an integration time nine times lower to obtain the same number of counts per channel than with the pixel-type system, although it is achieved at the expense of the spatial resolution.

These results demonstrate that we can perform beam monitoring, with different conditions of detection surface, dimensions, spatial resolution and acquisition time, with both microdetector systems. Additionally, the number of total counts will have also a direct impact on the values and the standard deviations of the physical microdosimetry quantities, as discussed below.

### Microdosimetry measurements

As explained above, and as indicated in the literature^14^, the lineal energy is calculated as the ratio between the energy deposited by the impinging particles in the SV of the microdetector ($\epsilon$) divided by its mean chord length (${\bar{l}}$). In the case of our experimental setup, with the direction of the proton beam perpendicular to the front face of the cylindrical detectors (Fig. 3b), the mean chord length corresponds to the thickness of the detectors, i.e., 19.86 $\mu$m. To obtain the lineal energy spectra, the output voltage pulse heights were stored as a list of events for each microdetector and irradiation energy as an HDF5 file. Then, data were properly converted to the corresponding energy spectra and analyzed by means of an in-house Python code and using the energy calibration described in 2.3 section. This code also allows us to generate the corresponding probability distributions of the lineal energy and the 2D maps of the relevant microdosimetry quantities. It takes less than one minute to process a file corresponding to a measurement with $\sim$10$^6$ events and allows us to treat the experimental data right after the irradiation. In this work, we focus on two of the most relevant microdosimetry physical parameters: the frequency-mean lineal energy (${\bar{y}}_F$) and the dose-average lineal energy (${\bar{y}}_D$). The values and distributions of both quantities were calculated by following the microdosimetry theory described by Rossi and Zaider^52^.

#### Microdosimetry spectra

**Figure 7:**
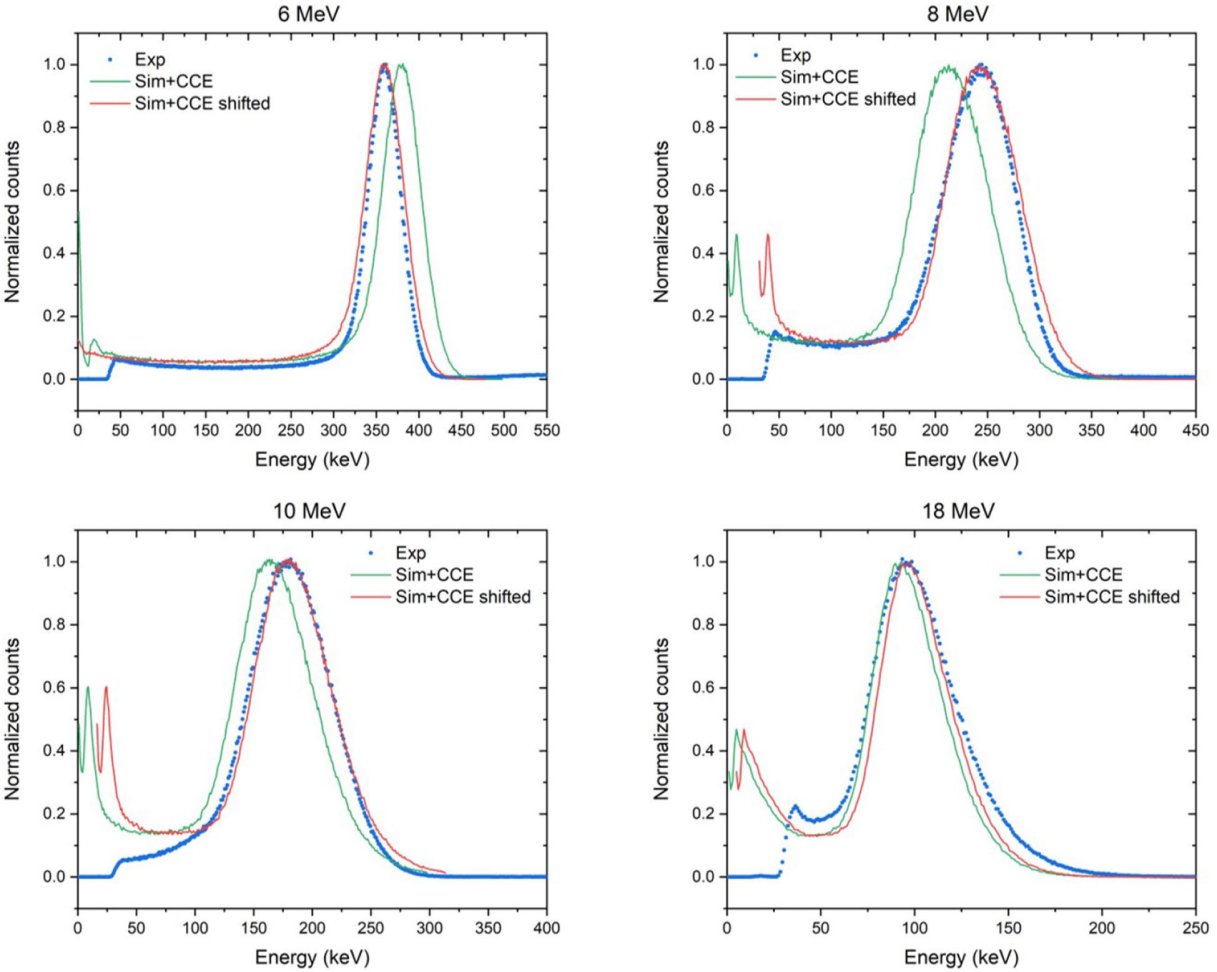
Experimental energy spectra (blue dots), Monte Carlo simulations of the spectra after applying the CCE correction (green solid lines), and simulated spectra when the corresponding shift offsets are applied (red solid lines) for 6, 8, 10 and 18 MeV proton beams.

The experimental energy spectra obtained from the 2 mm$\times$2 mm of the pixel-type system and from a 1 cm representative region of the pad-type system have been compared to Monte Carlo simulations. For the sake of simplification, only those corresponding to the first system, pixel-type, are shown. Similar tendencies were found for those corresponding to the pad-type system. Figure 7 shows the comparison between the experimental pulse-height spectra and those simulated once the CCE correction factor is applied^47^ for the four representative proton beam energies above. An energy shift between the experimental and the simulated peak maximum is observed in all the cases (from 4 to 30 keV). However, when a shift offset is applied, there is a very good agreement between both spectra shapes. These imbalances in the maximum peak may be due to unmanageable issues, e.g., uncertainties in the geometrical modeling of the beamline, imperfect multiple Coulomb scattering model in the Monte Carlo code, or to experimental misalignments since the daughterboard was placed manually. Another hypothesis is that those differences between simulated and measured spectra can be due to uncertainties in the energy calibration since it was not viable to perform it in vacuum neither at in a controlled environment (temperature and humidity), which may have an impact on the detector response. In addition, our energy calibration was performed with SRIM simulations, in order to have an independent intercomparision between the MC code used in the calibration and that used in the spectra comparison, but slightly different values of the deposited energy were found between SRIM and Geant4 simulations, which can also explain the shifts observed. Similar shifts have been previously found in microdosimetry studies at these micrometer scales^29,44^.

**Figure 8:**
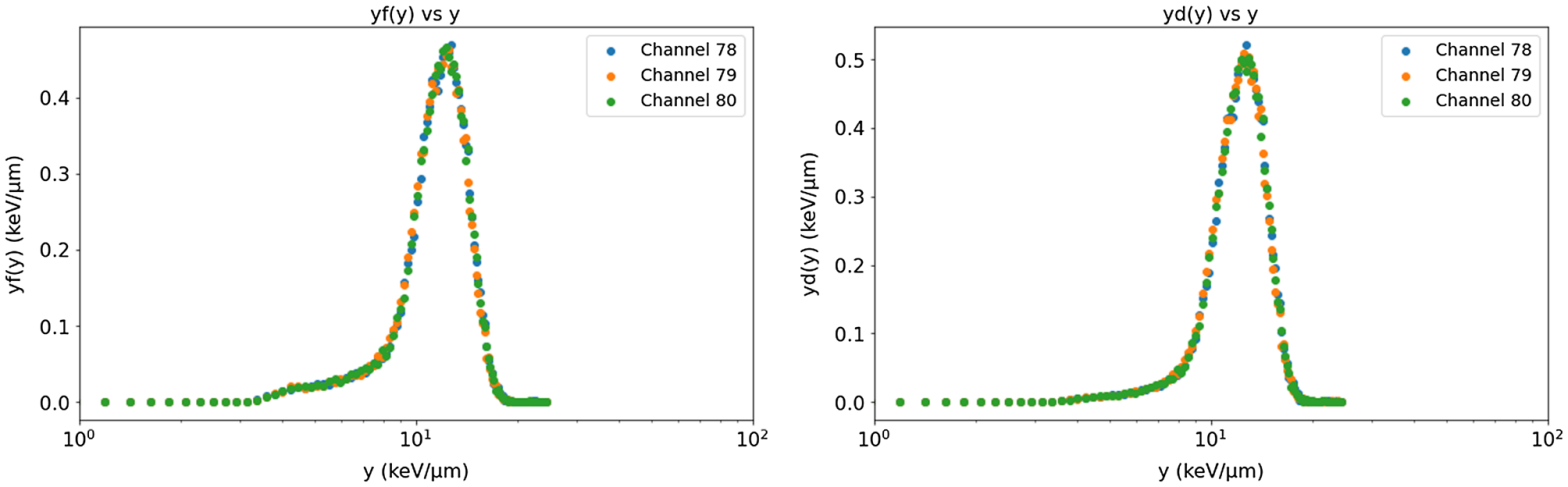
Lineal energy and absorbed dose distributions, *f*(*y*) and *d*(*y*), obtained in silicon with three different 3$\times$3 cells (each color in the graphs corresponds to one of the three cells) of the pad-type system under 8 MeV proton irradiation.

It is worth noting that, although we have used herein the events coming from multiple microdetectors, the present microdosimetry system and data analysis code allow to obtain the energy spectrum in each microdetector individually. Therefore, one could also obtain the probability distribution of the lineal energy *f*(*y*) and of the absorbed dose *d*(*y*) for each microdetector, i.e., with a spatial resolution of 200 $\mu m$ and 600 $\mu m$ for the pixel- and pad-type systems, respectively. This can be very useful for voxel-by-voxel RBE optimization with these resolutions, particularly in penumbras and out-of-field regions. As an example, Fig. 8 shows the probability distributions *f*(*y*) and *d*(*y*) (in silicon) for three different 3$\times$3 cells of the pad-type system for 8 MeV proton irradiation.

#### Frequency-mean lineal energy (${\bar{y}}_F$)

The lineal energy *y* is a stochastic quantity with a frequency (or probability) distribution *f*(*y*). The expected value of this distribution is the frequency-mean lineal energy (${\bar{y}}_F$), which is a non-stochastic quantity that can be calculated through Eq. 1.

1 $$\begin{aligned} {\bar{y}}_F=\int _{0}^{\infty } y\cdot f(y)dy \end{aligned}$$

In the case of our microdetector systems, we have obtained the ${\bar{y}}_F$ values for each individual channel (connected to one or nine detectors, depending on the system) using the distribution of the discrete events as expressed in Eq. 2, where *N* is the total number of events recorded by the channel.

2 $$\begin{aligned} {\bar{y}}_F=\sum _{i=1}^{N}y_i\cdot f(y_i) \end{aligned}$$

The standard deviation of the frequency-mean lineal energy ($\sigma {\bar{y}}_F$) has been obtained for each channel using Eq. 3.

3 $$\begin{aligned} \sigma {\bar{y}}_F=\sqrt{\frac{\displaystyle \sum _{i=1}^{N}(y_i-{\bar{y}}_F)^2}{N-1}} \end{aligned}$$

**Figure 9:**
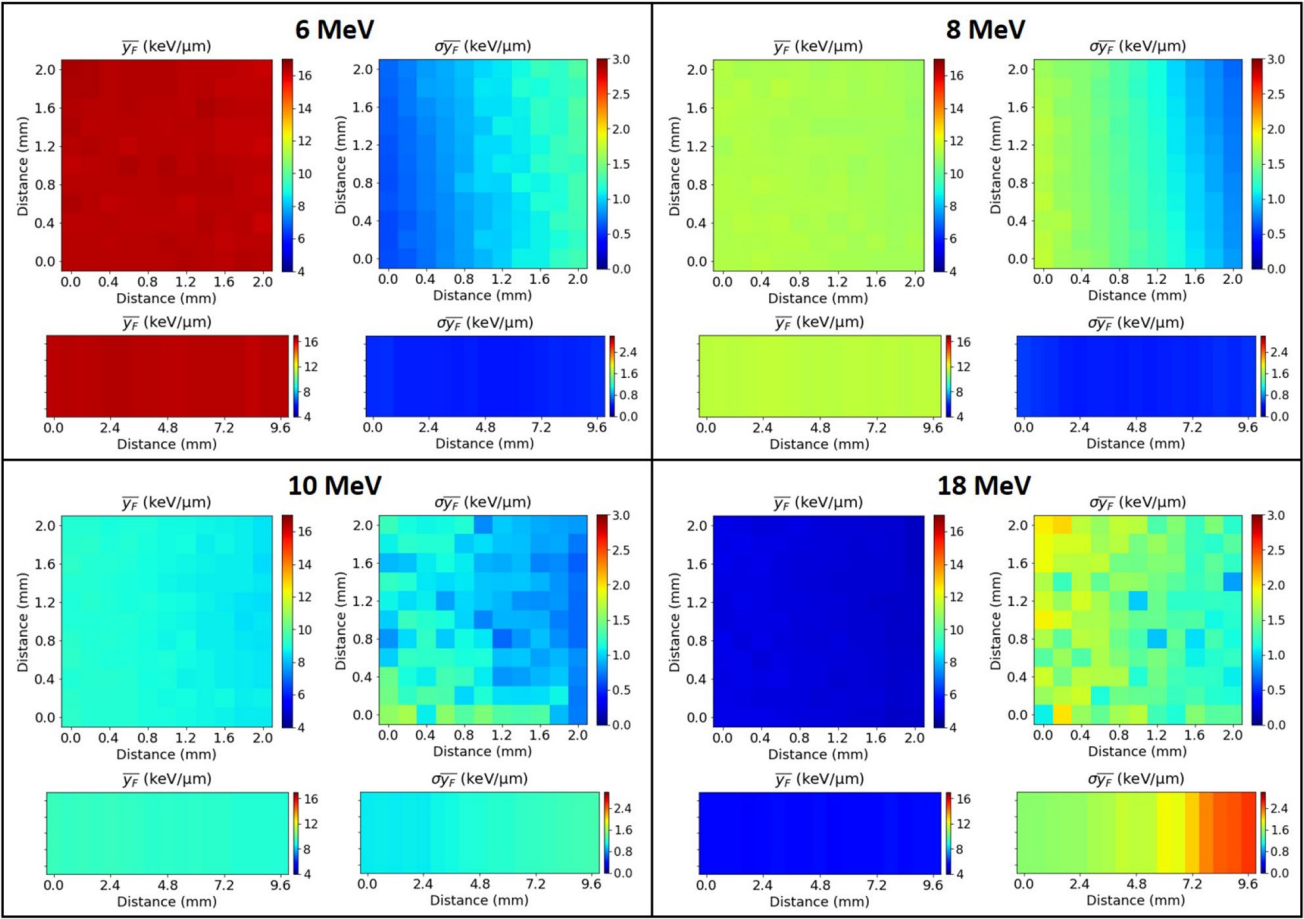
Results of ${\bar{y}}_F$ and $\sigma {\bar{y}}_F$ obtained with four representative energies. In each quadrant are depicted the results for the full pixel-type system (top) and for 1 cm representative region of the pad-type system (bottom).

In Fig. 9, each quadrant corresponds to one of the four beam energies discussed herein, and shows the frequency-mean lineal energy (left) and the corresponding standard deviations (right) for each channel of the pixel-type system (top) and for 1 cm of the pad-type system (bottom). For each beam energy we obtain, as expected, similar values of ${\bar{y}}_F$ for each pixel in both systems (with decreasing values of ${\bar{y}}_F$ for the increasing four beam energies), since this quantity depends only on the distribution of the energy deposited by the protons in the SV of the microdetectors. The standard deviation depends on the total number of counts measured by each channel as $1/\sqrt{N}$ (Eq. 3). As we had seen in Section 0.3, the number of counts was not the same for each microdetector, which explains the differences observed in the 2D maps of $\sigma {\bar{y}}_F$.

Table 1 summarizes the average of the ${\bar{y}}_F$ values, expressed as $<{\bar{y}}_F>$, and their standard deviation $\sigma {\bar{y}}_F>$, for the 121 channels (121 detectors) in the pixel-type system and for the 17 channels (153 detectors) in the 1 cm region of the pad-type system. We find differences in the average ${\bar{y}}_F$ values between both configurations ranging from 0.6 % and 9.8 % for the lowest and highest proton energies, respectively. This difference can be explained by the different surfaces covered by both systems and by the inhomogeneous distribution of the proton beam, due to the particle straggling produced in the beam when traveling through the kapton film and the 5.1 cm of air before arriving to the detection systems.

#### Dose-average lineal energy (${\bar{y}}_D$)

The absorbed dose distribution *d*(*y*) is given by Eq. 4.

4 $$\begin{aligned} d(y)= \frac{y\cdot f(y)}{{\bar{y}}_F} \end{aligned}$$

The expected value of *d*(*y*) is called the dose-mean lineal energy, ${\bar{y}}_D$, and is a non-stochastic quantity (Eq. 5).

5 $$\begin{aligned} {\bar{y}}_D&= \int _{0}^{\infty } y\cdot d(y) dy = \frac{1}{{\bar{y}}_F}\int _{0}^{\infty } y^2\cdot f(y) dy \end{aligned}$$

6 $$\begin{aligned} \sigma {\bar{y}}_D&=\sqrt{\frac{\displaystyle \sum _{i=1}^{N}(y_i-{\bar{y}}_D)^2}{N-1}} \end{aligned}$$

**Table 1:** Values of $<{\bar{y}}_F>$, $\sigma$ $_{<{\bar{y}}_F\!>}$, $<{\bar{y}}_D>$, and $\sigma$ $_{<{\bar{y}}_D\!>}$ obtained with both systems for four representative energies.

$E_{beam}$	$<{\bar{y}}_F>$ $\pm \sigma$ $_{<{\bar{y}}_F\!>}$ (keV/$\mu m$)			$<{\bar{y}}_D>$ $\pm \sigma$ $_{<{\bar{y}}_D\!>}$ (keV/$\mu m$)		
(MeV)	Pixel-type	Pad-type	Difference	Pixel-type	Pad-Type	Difference
6.0	16.40 ± 0.06	16.30 ± 0.04	0.6 %	17.25 ± 0.04	17.56 ± 0.07	1.8 %
8.0	11.31 ± 0.12	11.46 ± 0.05	1.3 %	12.25 ± 0.14	12.06 ± 0.05	1.6 %
10.0	8.88 ± 0.20	9.20 ± 0.12	3.5 %	9.45 ± 0.20	9.94 ± 0.22	5.1 %
18.0	5.12 ± 0.12	5.65 ± 0.04	9.8 %	5.59 ± 0.13	6.06 ± 0.07	8.1 %

The higher differences observed at 10 MeV and 18 MeV are due to the different threshold applied to both systems during the measurements because of their different level of noise. The threshold used for the pixel-type system was $\sim$ 34 keV, while for the pad-type system it was $\sim$ 65 keV. This produces more events at low energy for the pixel-type system, as it can be seen in Fig. 5, which are especially relevant for the spectra at 10 MeV and 18 MeV. If we do the analysis fixing a threshold of 65 keV for the pixel-type system, the differences found in the average ${\bar{y}}_F$ and ${\bar{y}}_D$ values of both systems are not higher than 5 %.

These results prove that, first, depending of the physical area to be analyzed, we may use either a two dimensional array or a linear one; second, we obtain a high reproducibility under similar experimental conditions with both array systems.

## Discussion

Although the beam energies used in this work (6–20 MeV) are below the energy range used in clinical proton therapy (70–200 MeV), this study is acceptable as a first approach considering that the proton energies are similar to those at the end of the distal edge in clinical scenarios. Nevertheless, it is worth noting that the low energy threshold obtained with this system (2 keV/$\mu$m) is limiting the measurement of low LET contributions, e.g., at the Bragg peak entrance of the high energy protons used in clinical scenarios.

Regarding the calibration procedure, in principle, it is expected to have a proportionality between voltage amplitude and ADC channels. The channel gain, as well as the linearity, are key parameters to calibrate both pixel and pad-type systems and obtain the corresponding experimental energy spectra. Every ROC has to be calibrated separately due to potential non-uniformities, e.g., ASIC specific effects. Additionally, the gain was obtained for each of the 128 readout channels of the corresponding ASICs. Hence, we have studied the deviation between the individual ROC channels and the global chain calibration to find the best performance of the whole sensor system. Low deviations between both methods were found, which makes possible the use of a calibration for the full chip rather than per channel in future systems of this type, for the sake of simplification. The maximum deviations of the gain values obtained with both methods were of 3.6 % and 6.7 % for the pixel- and pad-type systems, respectively, which shows that the gain between pixels is quite homogeneous. Potential gain variations between the channels of a ROC may contribute to the overall energy spread in radiation sensitive regions sharing large surfaces. The shifts in gain between ROCs might be caused by electronic or ASIC effects and, thus, they are unavoidable. Therefore, a careful calibration is required as large areas of these radiation detectors are covered. Additionally, a precise calibration should be made by using the same type of particle that those used in the measurements^51^. Otherwise, it could have an impact in the calibration curve and, consequently, in the final spectra. In order to avoid it, we performed a full energy calibration with the proton beams of ALTO using the minimum intervening materials between the particles and the detector.

Considering the beam monitoring, on the one hand, the results showed that the devices are able to obtain the beam profiles (intensity at each position) for beam currents between 100 pA and 200 pA with a resolution of 200 $\mu m$, which is a promising solution for high spatial resolution requirements. Furthermore, the 3Dcylindrical microsensors are manufactured over SOI wafers whose support may be selectively etched and obtaining ultra-thin sensors ($\le$ 20 $\mu m$). It avoids not only back-scattering contributions, but also reduces the possible scattering of the main beam, which is fundamental for minimizing the energy loss of the main particle beam. In the particular case of hadron therapy, the ion beams have currents (charge per unit time) in the order of tens of nA in proton and sub nA in carbon ion, but cover larger surfaces than those used during our measurements. Therefore, we chose to use beam currents of 100-200 pA in order to have ion fluence rates (number of particles per unit time per unit area) of the same order of magnitude than the ones used in this clinical application. On the other hand, depending on the detection surface and the spatial resolution required, the acquisition time can be reduced proportionally. For example, the acquisition time to accumulate the same statistics was 9 times lower in the pad-type system than in the pixel-type system, since it has 3$\times$3 detectors connected to each channel (instead of 1 detector per channel), at the cost of reducing the spatial resolution from 200 $\mu m$ to 600 $\mu m$. Likewise, since the number of total counts have a direct impact on the standard deviation values of the physical microdosimetry quantities, a trade-off must be found between the detection surface and the maximum available measurement time to reach suitable statistics. Interestingly, the diameter of the current 3D-microdetectors can be technologically reduced to a half or smaller (down to 9 $\mu m$), which would make possible to quadruple the beam intensity without saturation, i.e., reducing the possibility of pile-up in high flux environments. Additionally, no differences in the spectra were found due to potential radiation damage when same measurements were repeated, although further studies are planned to determine the average life of these devices working under common particle accelerator intensities. Furthermore, with our 3D architecture, the capacitance of a unitcell (i.e., a single sensitive volume or 3Dmicrodetector) is two orders of magnitude lower than a planar sensor of the same thickness^50^, which improves the overall signal-to-noise ratio.

With respect to the microdosimetry measurements, first, provided equal energy threshold in both pixel- and pad-type systems, the differences found in the ${\bar{y}}_F$ and ${\bar{y}}_D$ values are not higher than 5 %, thereby ensuring a low deviation between both systems. Second, current microdosimeters are mainly based on p-n planar junctions with different etched shapes around the sensitive volumes^28^. In contrast, our novel 3D-cylindrical architecture has a truly well-defined convex sensitive volume without charge sharing between adjacent electrodes. Third, we have customized a low-noise multi-channel readout electronics system to work at therapeutic fluence rates. Fourth, both the experimental ${\bar{y}}_F$ and ${\bar{y}}_D$ are in good agreement with the expected trends in the literature in the Bragg peak and distal edge (with energy values equivalent to those used herein) in clinical proton beams^29,58^ and radiology accelerator platforms^59^ with solid-state microdosimeters, and in a low-energy proton cyclotron^60^ with mini-TEPCs. For example, Pan *et al.*^60^ obtained an average ${\bar{y}}_D$ value of 6.4 keV/$\mu m$ (water-equivalent) irradiating with a 15 MeV proton beam at 50 mm from the cyclotron beam exit to the mini-TEPC (close to our 51 mm distance from the kapton layer in the beam exit to our microsensors). We obtained ${\bar{y}}_D$ values (in silicon) of 9.45 keV/$\mu m$ and 5.59 keV/$\mu m$ for 10 and 18 MeV proton energies (before the beam exit), respectively. If we apply a variable silicon-to-water conversion factor by using the ratio of the stopping power in both materials to these ${\bar{y}}_D$ values, we would get 6.62 and 4.19 keV/$\mu m$, respectively. It is worthwhile noting that the energy beam arriving to the internal sensitive volume of the Pan’s mini-TEPC is more slowed and broadened than that impinging into our microdetectors due to the wall-thickness/housing of that sensor. It means that the beam energy comparable to that used by Pan (i.e., 12.88 MeV as claimed by the authors) would be closer to 10 MeV, whose ${\bar{y}}_D$ value is 6.62 keV$\cdot$m$^{-1}$ in water-equivalent, which is in very good agreement with that obtained by Pan *et al.*

Finally, it was observed that the general spectra shapes of the experimental results were reproduced by those simulated with the Monte Carlo used. However, systematic energy shifts (from 4 to 30 keV) are found between the experimental and the simulated peak maxima. Nevertheless, when a shift offset is properly applied in energy range, there is an overall good agreement between the experimental and simulated spectra. These shifts are likely due to unmanageable inaccuracies associated to the modeling and they have been previously found in the literature at the micrometer scale^29,44^.

## Conclusion

We present the first microdosimeter layouts that allow us to generate a modular structure to scale the sensitive areas towards the centimeter scale finding a trade-off between a doable multi-channel electronics and the minimum spatial resolution required depending on the final application. Two scalable microdetector arrays for both beam monitoring and microdosimetry purposes have been manufactured and characterized using proton beams at different energies with clinical-equivalent fluence rates.

The present paper reports on the first comparison between the two microdosimeter systems, having different geometries and 2 mm$\times$2 mm and 0.4 mm$\times$12 cm detection surfaces, under irradiation with protons with the highest spatial resolution so far. The new 3D-cylindrical microdetector arrays can be used not only for microdosimetry measurements, but also for beam monitoring with high spatial resolution.

Finally, it is important to remark that, with this type of microsensors, we could obtain the microdosimetry distributions in two dimensions in more complex clinical conditions (e.g., in heterogeneous LET distributions typically occurred at the tumor boundaries that can be critical for organs-at-risk), with a spatial resolution of 200 $\mu m$ and 600 $\mu m$, or lower by adjusting the layout. It could be used for further voxel-by-voxel RBE optimization (with these resolutions). Therefore, these large area sensors can also be applied in hadron therapy to assess the microdosimetry distributions in critical areas, e.g., organs-at-risk and penumbras, as well as out-of-field regions.
